# Exploring complications following cranioplasty after decompressive hemicraniectomy: A retrospective bicenter assessment of autologous, PMMA and CAD implants

**DOI:** 10.1007/s10143-024-02309-z

**Published:** 2024-01-29

**Authors:** A. Pfnür, D. Tosin, M. Petkov, O. Sharon, B. Mayer, C. R. Wirtz, A. Knoll, A. Pala

**Affiliations:** 1 Department of Neurosurgery, University of Ulm, Albert-Einstein-Allee 23, 89081 Ulm, Germany; 2 Department of Neurosurgery, University of Ulm, Lindenallee 2, 89312 Günzburg, Germany; 3 Institute of Epidemiology and Medical Biometry, University of Ulm, Schwabstraße 13, 89075 Ulm, Germany

**Keywords:** Cranioplasty, Decompressive hemicraniectomy, Complications

## Abstract

Cranioplasty (CP) after decompressive hemicraniectomy (DHC) is a common neurosurgical procedure with a high complication rate. The best material for the repair of large cranial defects is unclear. The aim of this study was to evaluate different implant materials regarding surgery related complications after CP. Type of materials include the autologous bone flap (ABF), polymethylmethacrylate (PMMA), calcium phosphate reinforced with titanium mesh (CaP-Ti), polyetheretherketone (PEEK) and hydroxyapatite (HA). A retrospective, descriptive, observational bicenter study was performed, medical data of all patients who underwent CP after DHC between January 1st, 2016 and December 31st, 2022 were analyzed. Follow-up was until December 31st, 2023. 139 consecutive patients with a median age of 54 years who received either PMMA (56/139; 40.3%), PEEK (35/139; 25.2%), CaP-Ti (21/139; 15.1%), ABF (25/139; 18.0%) or HA (2/139; 1.4%) cranial implant after DHC were included in the study. Median time from DHC to CP was 117 days and median follow-up period was 43 months. Surgical site infection was the most frequent surgery-related complication (13.7%; 19/139). PEEK implants were mostly affected (28.6%; 10/35), followed by ABF (20%; 5/25), CaP-Ti implants (9.5%; 2/21) and PMMA implants (1.7%, 1/56). Explantation was necessary for 9 PEEK implants (25.7%; 9/35), 6 ABFs (24.0%; 6/25), 3 CaP-Ti implants (14.3%; 3/21) and 4 PMMA implants (7.1%; 4/56). Besides infection, a postoperative hematoma was the most common cause. Median surgical time was 106 min, neither longer surgical time nor use of anticoagulation were significantly related to higher infection rates (*p* = 0.547; *p* = 0.152 respectively). Ventriculoperitoneal shunt implantation prior to CP was noted in 33.8% (47/139) and not significantly associated with surgical related complications. Perioperative lumbar drainage, due to bulging brain, inserted in 38 patients (27.3%; 38/139) before surgery was protective when it comes to explantation of the implant (*p* = 0.035). Based on our results, CP is still related to a relatively high number of infections and further complications. Implant material seems to have a high effect on postoperative infections, since surgical time, anticoagulation therapy and hydrocephalus did not show a statistically significant effect on postoperative complications in this study. PEEK implants and ABFs seem to possess higher risk of postoperative infection. More biocompatible implants such as CaP-Ti might be beneficial. Further, prospective studies are necessary to answer this question.

## Introduction

The reconstruction of large cranial bone defects results in restoration of cerebral protection and the shape of the neurocranium. Cranioplasty (CP) can improve cognitive and motor functions. It improves circulatory disorders and insufficient blood supply and restores the distribution of solutes in cerebrospinal and interstitial fluid, which is impaired after decompressive hemicraniectomy (DHC) [1, 2]. CP can prevent and treat hydrocephalus in patients after DHC [3].

It has been the subject of numerous technical and material advancements, owing to the complexity and diversity of conditions it addresses. Despite these advancements, it continues to pose significant challenges, especially related to optimal timing for surgery and implant material selection. Most importantly, even though CP is a common neurosurgical procedure, it still shows a high complication rate around 31%, while mortality rate is reported to be 3% [4].

Common complications include surgical site infection (SSI), epidural hematoma, seizures, malignant cerebral edema, implant dislocation as well as bone flap resorption. Along with the frequent complications comes a surgical revision rate of 14% [5]. This underlines the need for continual reassessment and advancement of current surgical practices and materials used. Despite growing research on this topic, still lots of open questions regarding the optimal timing and the best material for CP remain without a widely accepted answer. Additionally, different institutions may often use different practices, as there is yet no consensus on a standard of care for CP. CP within 4 weeks after initial craniectomy is called ultra-early and could be beneficial for neurological outcomes, but may pose a higher risk for SSI compared to an early approach within 12 weeks [6–8]. Although CP using the autologous bone flap (ABF) remains a commonly chosen technique, it is discussed controversially due to the well documented risk of bone flap resorption [9–11]. As an alternative, various allogenic implants are used in clinical practice such as polymethylmethacrylate (PMMA), polyetheretherketone (PEEK), hydroxyapatite (HA) and titanium-reinforced calcium phosphate (CaP-Ti). Single materials differ in their strength, thermal properties, biocompatibility and radiographic features, while also related costs for health institutions vary sensibly [12, 13]. Nevertheless, previous works comparing different implant materials with respect to surgical complications often came to contradictory conclusions, so that there is still no consensus on the best material for CP [14, 15]. We have performed a retrospective analysis of CP after decompressive hemicraniectomy (DHC) including different materials in two hospitals. The focus of the study was postoperative complications, since these not only increase the morbidity and mortality but also extend hospital stays and the overall healthcare costs.

## Materials and methods

### Patient selection

A retrospective, descriptive, observational bicenter study was performed at our institutions in Günzburg and Ulm including 139 patients who underwent CP after DHC between January 1st, 2016 and December 31st, 2022. Cases of bifrontal craniectomy were excluded from data collection. Data regarding postoperative complications were collected until December 31st, 2023. All patients were at least 18 years old at the date of CP. Different implants including autologous bone, PMMA, CaP-Ti, HA and PEEK based computer aided design (CAD) implants were used over the years and analyzed for this study. Besides the implant material, age, BMI, surgery related complications, time of surgery, anticoagulation therapy, known risk factors for infections and type of artificial dura were analyzed.

### Variable definition

Anticoagulation and antiplatelet therapy included oral anticoagulants, therapeutic dose of low molecular heparin, acetylsalicylic acid, clopidogrel and prasugrel. Both anticoagulants and antiaggregants were paused prior to CP as part of our standard practice, while therapy with acetylsalicylic acid might have been continued depending on its indication. Only surgical complications, namely those which lead to a surgical revision, were assessed for the purpose of this analysis. SSI and wound dehiscence were considered as a single complication under the definition of infection. We termed reoperation as any surgery performed after the CP to treat complications.

### Surgical technique and patient management

We included patients receiving CP after DHC due to malignant stroke, traumatic brain injury, spontaneous subarachnoid hemorrhage and intracerebral hemorrhage. Regarding DHC, a large frontotemporoparietal craniectomy as previously described was performed [16, 17]. CP was scheduled about 3 months after DHC, allowing the patient and surgical site to completely heal prior to repair of the bone defect. In a few selected cases, CP was planned and performed earlier due to other patient-related factors or comorbidities, such as sinking skin flap syndrome. In case of severe brain bulging, a lumbar drainage was inserted the day before cranioplasty surgery.

ABFs were stored in an -80-degree Celsius freezer. The bone was fixed to the skull using titanium plates and screws. PMMA implants were either shaped during surgery in free hand technique or before surgery using a cast obtained previously from the patient’s ABF. The latter technique was chosen preferably for PMMA cranioplasties after DHC. Synthetic patient-specific implants (PSI) for cranioplasty were designed using 1.0 mm thick slices of a CT scan. The design was confirmed by a surgeon in our department before production. CaP-Ti implants are composed of calcium phosphate in the form of hexagonal tiles reinforced by a titanium mesh and were bought from OssDsign (Uppsala, Sweden). PEEK implants are a polyaromatic semi-crystalline thermoplastic polymer and were bought from Evonos (Tuttlingen, Germany). Nonabsorbable ceramic porous hydroxyapatite (HA) implants were bought from Finceramica (Faenza, Italy).

CaP-Ti implants were soaked in a 5% gentamicin solution prior to fixation to the cranial defect [18]. On the first postoperative day, a CT scan was performed on each patient to evaluate complications and the implant fit. Most patients were reevaluated in our outpatient department, but there was no routine follow-up planned for patients after cranioplasty.

### Statistical analysis

Statistical analysis was performed using SPSS 26.0 (Lead Technologies, Inc.; Charlotte, USA). Descriptive statistics as well as Kruskal–Wallis-Test were used for the analysis. Univariate and multivariable regression models for SSI, surgical revision and explantation were calculated. Influencing variables were age, BMI, surgical time, lumbar drain during the surgery, type of implant and the time between DHC and CP. All variables which achieved significance in univariate analysis were included in multivariate analysis. A *p* value < 0.05 was considered statistically significant. Findings were reported as median. GraphPad Prism 9.0 (GraphPad Software, Inc.; Boston, USA) was used to create figures.

## Results

### Baseline data

A total of 139 patients were included in our retrospective analysis. Baseline data of our patient population assessed at time of CP, including initial diagnoses leading to DHC and comorbidities, are listed in Table 1. Here we compare the characteristics of the whole population and of the single subgroups according to the chosen implant material for CP.

**Table 1:** Baseline data at time of CP. *^1^ Other diagnoses included meningoencephalitis, cerebral venous sinus thrombosis and posterior reversible encephalopathy syndrome leading to diffuse cerebral edema. Age, BMI and the ability of a patient to walk were assessed at the time of CP

Baseline data	Overall	ABFs	PMMA	PEEK	CaP-Ti	HA
Study population (no.)	139 (100%)	25/139 (18.0%)	56/139 (40.3%)	35/139 (25.2%)	21/139 (15.1%)	2/139 (1.4%)
Median age (years)	54	54	53	58	45	55
Diagnosis						
- Stroke	56/139 (40.3%)	8/25 (32.0%)	27/56 (48.2%)	11/35 (31.4%)	9/21 (42.9%)	1/2 (50.0%)
- TBI	41/139 (29.5%)	9/25 (36.0%)	16/56 (28.6%)	9/35 (25.7%)	7/21 (33.3%)	0
- SAH/ICH	33/139 (23.7%)	7/25 (28.0%)	10/56 (17.8%)	11/35 (31.4%)	4/21 (19.0%)	1/2 (50.0%)
- Other*^1^	9/139 (6.5%)	1/25 (4.0%)	3/56 (5.4%)	4/35 (10.5%)	1/21 (4.8%)	0
VPS prior to CP	47/139 (33.8%)	9/25 (36.0%)	13/56 (23.2%)	17/35 (48.6%)	7/21 (33.3%)	1/2 (50.0%)
Unable to walk	82/139 (59.0%)	13/25 (52.0%)	29/56 (51.8%)	29/35 (82.9%)	9/21 (42.9%)	2/2 (100%)
Median BMI (kg/m^2^)	25	27	25.5	24	25	25
Comorbidities						
- Obesity	18/139 (12.9%)	2/25 (8.0%)	18/56 (32.1%)	3/35 (8.6%)	3/21 (14.3%)	0
- Diabetes	9/139 (6.5%)	2/25 (8.0%)	9/56 (16.1%)	1/35 (2.9%)	1/21 (4.8%)	0
- Coronary artery disease	11/139 (7.9%)	1/25 (4.0%)	4/56 (7.1%)	7/35 (20.0%)	0	
- Alcohol abuse	18/139 (12.9%)	3/25 (12.0%)	6/56 (10.7%)	5/35 (14.3%)	4/21 (19.0%)	0
- Smoking	28/139 (20.1%)	5/25 (20.0%)	12/56 (21.4%)	10/35 (28.6%)	4/21 (19.0%)	0
Anticoagulant or antiplatelet therapy	41/139 (29.5%)	7/25 (28.0%)	12/56 (21.4%)	14/35 (40.0%)	10/21 (47.6%)	1/2 (50.0%)
Median time to CP (days)	117	108	94	146	136	226
Median surgical time (minutes)	106	174	98	87	108	168
Perioperative lumbar drain	38/139 (27.3%)	6/25 (24.0%)	23/56 (41.1%)	3/35 (8.6%)	6/21 (28.6%)	0
Median follow-up (months)	43	70	75	24	16	70

**Fig. 1:**
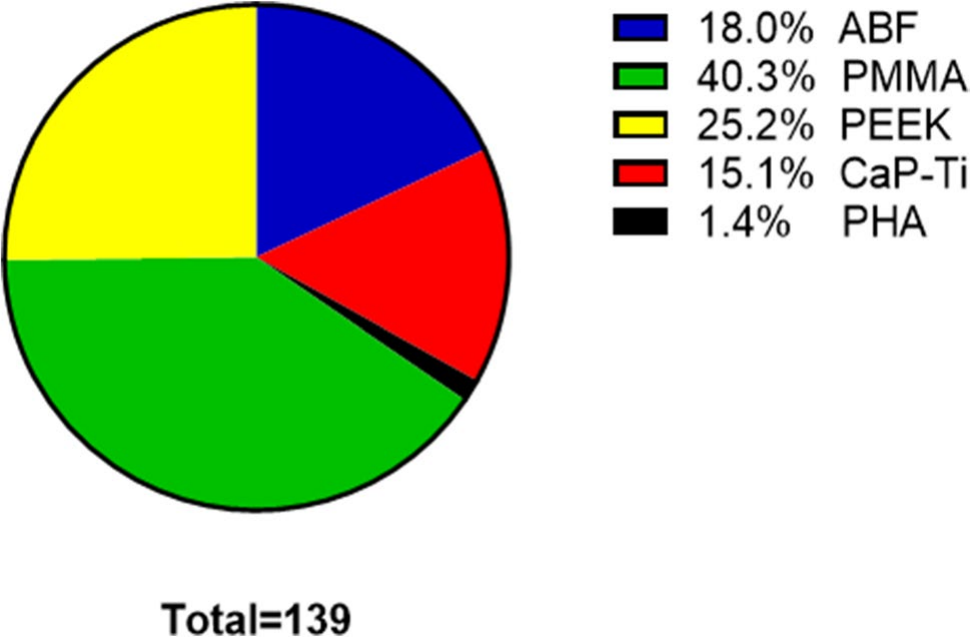
Proportions of single implant materials related to the total of cranioplasties performed

Single subgroups were in decreasing size order PMMA implants with 56/139 cases (40.3%), PEEK with 35/139 cases (25.2%), ABF with 25/139 cases (18.0%) and CaP-Ti with 21/139 cases (15.1%). Since only 2/139 patients (1.4%) received HA implants, results concerning this subgroup are only illustrated in Table 1 and discussed below, but will not be described in detail in this section (Fig. 1).

Stroke was the most common condition leading to initial craniectomy in our study population, followed by TBI and SAH, as well as ICH. Less frequent diagnoses included diffuse cerebral edema (9/139, 6.5%) due to cerebral venous sinus thrombosis, meningoencephalitis and posterior reversible encephalopathy syndrome. The majority of patients in the PMMA (27/56, 48.2%) and CaP-Ti (9/21, 42.9%) subgroups also suffered stroke as initial diagnosis, while stroke was as frequent as SAH/ICH in the PEEK subgroup (11/35, 31.4%). In the ABF subgroup instead, TBI resulted to be the most common primary condition (9/25, 36.0%). Sinking skin flap syndrome after DHC was diagnosed in 9/139 cases (6.5%). Median follow-up time after CP amounted to 43 months.

### Cranioplasty

Median age at time of CP was 54 years in the overall patient population, without any relevant differences among the single subgroups except for CaP-Ti patients having the youngest median age of 45 years. The PEEK subgroup showed the oldest median age of 58 years. Median time between initial surgery and CP was 117 days. In the PMMA subgroup, median time to CP was the shortest (94 days) and it was the longest in the PEEK subgroup with 146 days. Median surgical time in the whole study population was 106 min. CP using ABFs required the longest median time of 174 min, while surgical time was similarly shorter among the other subgroups, with PEEK cranioplasties requiring the shortest median time (87 min). A lumbar drain was perioperatively placed in 38/139 patients (27.3%), with little differences between subgroups except of PEEK with only 3/35 lumbar drains (8.6%).

### Surgical complications

For the purpose of the present analysis, SSI was defined as any wound dehiscence, abscess or epidural empyema that occurred after CP. SSI was the most frequent complication in our cohort with 19/139 affected patients (13.7%). Explantation of the cranial implant was the most frequent (24/139, 17.3%) form of reoperation performed. An additional 5/139 (3.6%) patients developed a persistent subdural hygroma or subcutaneous CSF collection, which led to explantation of the cranial implant as well. The second most frequent indication for surgical revision was subdural/epidural hematoma evacuation (12/139, 8.6%). Another 5/139 patients (3.6%) had to undergo refixation of a displaced implant.

Two patients of 139 (1.4%) suffered diffuse cerebral edema after CP with lethal outcome. One patient died due to a severe postoperative intracerebral bleeding after CP. In cases suffering surgical complications, median time between CP and explantation surgery was 37 days and median time until reoperation was 30 days. Table 2 illustrates the most common surgical complications in our patient population and in subgroups sorted by CP materials.

**Table 2:** Surgical complications. *^1^ Indications for reoperation included SSI, subdural/epidural hematoma, implant displacement, cerebrospinal fluid collection and subdural hygroma. Overall reoperation comprises cases of complication treatment involving as well as not involving explantation of the cranial implant

Surgical complications	Overall	ABFs	PMMA	PEEK	CaP-Ti	HA
SSI	19/139 (13.7%)	5/25 (20.0%)	1/56 (1.7%)	10/35 (28.6%)	2/21 (9.5%)	1/2 (50.0%)
Explantation	24/139 (17.3%)	6/25 (24.0%)	4/56 (7.1%)	9/35 (25.7%)	3/21 (14.3%)	2/2 (100.0%)
Overall reoperation*^1^	40/139 (28.8%)	12/25 (48.0%)	8/56 (14.3%)	14/35 (40.0%)	4/21 (19.0%)	2/2 (100.0%)

### Surgical site infection

SSI occurred most frequently in the PEEK subgroup with 10/35 cases (28.6%), followed by ABF (5/25, 20.0%), CaP-Ti (2/21, 9.5%) and PMMA (1/56, 1.7%). In univariate analysis, we found a statistically significant association between PEEK material and SSI (*p* = 0.005, OR = 2.054). Moreover, a higher BMI (*p* = 0.003, OR 0.799) and PMMA implants showed a significant association with no SSI (*p* = 0.009, OR 0.066). Besides, an older age at time of CP seemed to be weakly associated with SSI, but without reaching statistical significance (*p* = 0,070). In multivariate analysis, low BMI was the only factor significantly associated with SSI (*p* = 0.007, HR 0.788). No association with SSI was found for other implant materials, patient’s age, surgical time, preoperative lumbar drain, anticoagulant or antiplatelet therapy at time to CP and VPS prior to CP.

### Explantation

We found the highest explantation rate in the PEEK (9/35, 25.7%) and ABF (6/25, 24.0%) subgroup, followed by CaP-Ti (3/21, 14.3%) and PMMA (4/56, 7.1%). Preoperative insertion of a lumbar drain in patients presenting brain bulging over the edge of the cranial defect resulted as a protective factor against explantation (*p* = 0.035, OR 5.013) in univariate analysis. Out of 38 patients perioperatively treated with lumbar drain, 2 underwent explantation (5.3%). Among the remaining 101 patients, another 22 implants had to be removed (21.8%). BMI, age, surgical time, anticoagulant or antiplatelet therapy, time to CP and VPS prior to CP were not found to be associated with explantation.

### Reoperation

**Fig. 2:**
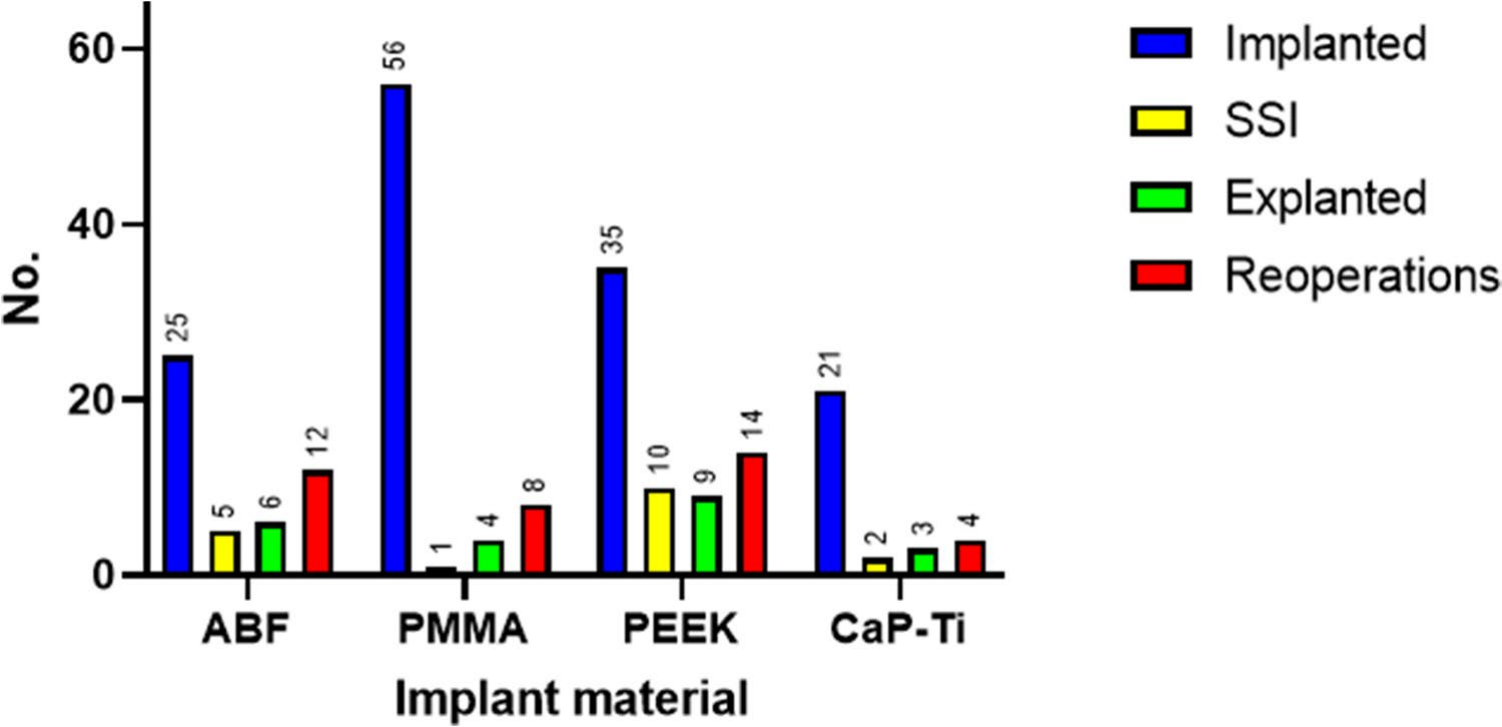
Surgical complications in relation to implant materials. HA cases are not shown here to simplify the diagram

In our whole study population, we registered 40/139 (28.8%) cases of reoperation. The ABF subgroup had the highest reoperation rate (12/25, 48.0%), followed by the PEEK (14/35, 40.0%), CaP-Ti (4/21, 19.0%) and PMMA (8/56, 14.3%) subgroups (Fig. 2). Reoperation was not significantly associated with age, surgical time, perioperative treatment with lumbar drain, body mass index, time to CP and anticoagulant or antiplatelet therapy.

## Discussion

### Complications related to implant materials

PEEK implants together with ABFs showed the highest rate of SSI. Similarly, PEEK implants were related to the highest rate of explantation. Revision surgery was most common after cranioplasty using ABFs followed by PEEK implants. In the literature, there are conflicting reports regarding cranioplasty with PEEK. In a meta-analysis from Henry et al. PEEK had the lowest infection rate with only 5% in 157 cases, compared to PMMA, HA and Ti [5]. In another retrospective study, 12.5% of patients treated with PEEK implants suffered infection compared to 25% in Ti implants [19]. Other authors report relative high explantation rates in association to PEEK. The overall complication rate of PEEK cranioplasty was 28% in a study from Jonkergouw et al. in 40 cases (13%) due to infection [20]. An even higher infection rate (27.8%) is mentioned in a study from Rosinski et al. regarding custom PEEK implants compared to Ti implants [21]. PEEK is a hydrophobic and bioinert material, so adhesion of osteoblasts to its surface and therefore osteoblastic differentiation and proliferation are prevented. After surgery, the implant is not incorporated in the surrounding cranium bone [22, 23]. Furthermore, other authors stated that the lack of osseointegration in PEEK can lead to issues like implant migration and infection [24]. It also needs to be noticed that our PEEK subgroup included the highest percentage of smokers (28.6%), patients unable to walk (82.9%) and of those treated with VP shunt prior to CP (48.6%) compared to the other subgroups excluding HA. These single features could be related to poor wound healing and thus contribute to the high rates of SSI and explantation in the PEEK subgroup, even though they were not significantly associated with any surgical complication in univariate analysis.

In our whole study population, PMMA followed by CaP-Ti implants had the lowest SSI, explantation and revision surgery rates in comparison to other cranioplasty materials. In a large retrospective series with 139 patients receiving PMMA, infection rate was very high with 42.5% [25]. In another study, revision surgery for PMMA was 22.9% [26]. As described before, reported differences in CP materials were often contradicted by other studies [27].

Our two centers used to perform CP with PMMA implants during the earlier years considered for this data collection, while custom-made implants were introduced more recently in our clinical practice. Thus, the longer experience with PMMA might explain the relatively low complication rate in our study. Furthermore, median time to CP was the lowest for PMMA implants (94 days) compared to the other subgroups. Earlier cranioplasty has been suggested as a potential factor reducing postoperative complications in previous works [28].

PMMA implants were produced in-house, either hand-shaped during surgery or shortly before by using a cast of the same patient’s explanted autologous bone flap. Conversely, there might be delays in scheduling cranioplasties with patient-specific implants due to the ordering, production and shipment process, which possibly explains the longer time to CP in the PEEK and CaP-Ti subgroups. However, we did not observe a significant association between time to CP and surgical complications in our study.

CaP-Ti implants showed the second lowest rates of SSI, explantation and overall revision surgeries. A possible explanation for lower complication rates with CaP-Ti implants could be that CaP promotes neovascularization and osseointegration. Sundblom et al. identified vascularized autologous bone formation in CaP implants in 4 patients, 5–38 months after implantation [29]. The bone ingrowth from boundaries of the cranial defect could prevent atrophy and poor wound healing [30, 31] and it may even become possible to treat existing implant infection with systemic antibiotics without removal of the implant if vascularized bone integration has occurred. Moreover, an in vitro study from Sundblom and colleagues analysed local drug release after soaking different material samples in a 200 μg/ml and a 400 μg/ml gentamicin solution at room temperature for 15 min. CaP samples could uptake gentamicin and release it over time, while Ti and PEEK showed no gentamicin uptake and consequent release [18]. Considering that we soaked all CaP-Ti implants in gentamicin solution, it might be another factor contributing to the relatively low SSI and explantation rates. CaP-Ti implants were introduced later in our two institutions and were initially chosen specifically for patients with a supposedly complicated surgical site, for instance those showing a stiff scar tissue or a sinking skin flap. The lower level of experience with CaP-Ti and a possible selection bias might hence contribute to surgical complications in this subgroup. Only in the recent three years, CaP-Ti became the most frequently used implant material in our routine cranioplasties.

HA implants show similar biological properties, it supports bone ingrowth and osseointegration [32, 33]. In our retrospective study, there were only two patients with HA implants included. Therefore, it is not possible to draw conclusions about the relation between HA implants and postoperative complications in this study. A recent multicenter study by Zaed et al. focusing on cranioplasty with HA reported 4.86% infections and 3.64% explantations [34]. The same authors also found 0.85% cases of implant fracture, as HA requires osseointegration before it can become resistant. Another study reported a higher fracture rate (20.8%) [35]. Henry and colleagues reported in their metaanalysis a revision surgery rate of 12% for HA [5].

### Potential risk factors for surgical complications

Additionally, we evaluated other possible risk factors resulting in explantation. In our study, perioperative placement of a lumbar drain for CSF drainage has been significantly associated with lower rates of explantation. CSF drainage before or during cranioplasty can be necessary in patients with a bulging brain parenchyma to allow fixation of the implant. Inserting a lumbar drain before performing surgery seems to be safer than intraoperative puncture of ventricles, as the latter is related to a damage of brain parenchyma and might lead to intracerebral haemorrhage [36]. Interestingly, there is growing evidence that lumbar drainage after hemicraniectomy can prevent infections and wound healing complications [37].

However, there are reported cases where a lumbar drain in patients after decompressive hemicraniectomy supposedly lead to paradoxical brain herniation. In theory, decreasing brain swelling leads to a higher difference between atmospheric pressure and intracranial pressure. It is a very rare complication but can prove lethal [38, 39]. Thus, there is a need to monitor patients treated with lumbar drain closely. In this regard, some authors suggest intracranial and lumbar pressure monitoring [40].

Our results also suggest that lower BMI at time of CP is associated with an increased risk for SSI. Baseline data indicate little difference of median BMI in the single patient subgroups sorted by implant material. Bedridden patients or those suffering from severe neurologic deficits because of their primary diagnosis often experience a relevant weight loss as far as cachexia due to malnutrition. Cachexia and malnutrition are known to impair wound healing [41]. These additional factors might act as confounders in the relation between BMI and SSI. We did not find a similar relation between obesity at the time of CP and SSI in our analysis.

## Limitations

This study is limited due to the retrospective, bicenter design and drawing definite conclusions from results with a small number of patients should be done carefully. This analysis lacks a standardised follow-up strategy and complication assessment relies on the evaluation of patient readmissions at our institution, therefore some complications might be overseen and their total amount underestimated. Even though follow-up was heterogeneous among different patient subgroups, the shortest median follow-up time still amounted to more than one year (CaP-Ti subgroup: 16 months).

Data collection might also be subject to selection bias. In particular, as the majority of our cases (40.3%) underwent DHC following stroke, our patient sample differs from other published cohorts presenting TBI as the main indication for DHC [42–44]. As a consequence, our results should be compared to those of other analyses only after considering the composition of the respective patient samples in terms of primary diagnoses leading to DHC. Another major limitation of our study is the relatively small patient population, which might impair the identification of factors associated with complications after CP.

## Conclusion

Based on our results, CP is still related to a relatively high number of infections and further postoperative complications. The choice of an implant material for cranioplasty seems to have a relevant impact on surgical complication rates. Especially PEEK and ABF seem to be associated with higher complication rates, in particular with SSI. On the other hand, surgical time, anticoagulation, antiplatelet therapy and hydrocephalus did not show a statistically significant association to postoperative complications in our study. More biocompatible implants such as CaP-Ti might be beneficial in terms of a reduced risk of postoperative complications. Prospective randomized trials are necessary to further investigate this matter.

## Data Availability

All data are available from the corresponding author on reasonable request.
